# The Alcohol Use Disorders Identification Test (AUDIT): reliability and validity of the Greek version

**DOI:** 10.1186/1744-859X-8-11

**Published:** 2009-05-14

**Authors:** George Moussas, Georgia Dadouti, Athanassios Douzenis, Evangelos Poulis, Athanassios Tzelembis, Dimitris Bratis, Christos Christodoulou, Lefteris Lykouras

**Affiliations:** 1 Second Psychiatry Department Athens University Medical School, Attikon Hospital, Athens, Greece; 2 Athens Psychiatric Hospital, Alcohol Detoxification and Short Term Treatment Unit, Athens, Greece; 3 Psychiatric Department, Sotiria General Hospital, Athens, Greece

## Abstract

**Background:**

Problems associated with alcohol abuse are recognised by the World Health Organization as a major health issue, which according to most recent estimations is responsible for 1.4% of the total world burden of morbidity and has been proven to increase mortality risk by 50%. Because of the size and severity of the problem, early detection is very important. This requires easy to use and specific tools. One of these is the Alcohol Use Disorders Identification Test (AUDIT).

**Aim:**

This study aims to standardise the questionnaire in a Greek population.

**Methods:**

AUDIT was translated and back-translated from its original language by two English-speaking psychiatrists. The tool contains 10 questions. A score ≥ 11 is an indication of serious abuse/dependence. In the study, 218 subjects took part: 128 were males and 90 females. The average age was 40.71 years (± 11.34). From the 218 individuals, 109 (75 male, 34 female) fulfilled the criteria for alcohol dependence according to the Diagnostic and Statistical Manual of Mental Disorders, 4th edition (DSM-IV), and presented requesting admission; 109 subjects (53 male, 56 female) were healthy controls.

**Results:**

Internal reliability (Cronbach α) was 0.80 for the controls and 0.80 for the alcohol-dependent individuals. Controls had significantly lower average scores (t test *P *< 0.001) when compared to the alcoholics. The questionnaire's sensitivity for scores >8 was 0.98 and its specificity was 0.94 for the same score. For the alcohol-dependent sample 3% scored as false negatives and from the control group 1.8% scored false positives. In the alcohol-dependent sample there was no difference between males and females in their average scores (t test *P *> 0.05).

**Conclusion:**

The Greek version of AUDIT has increased internal reliability and validity. It detects 97% of the alcohol-dependent individuals and has a high sensitivity and specificity. AUDIT is easy to use, quick and reliable and can be very useful in detection alcohol problems in sensitive populations.

## Introduction

Problems associated with alcohol abuse are recognised by the World Health Organization (WHO) as a major health issue, which according to most recent estimations is responsible for 1.4% of the total world burden of morbidity [1]. In the US alone, 8 million individuals aged 18 or more fulfil Diagnostic and Statistical Manual of Mental Disorders, 4th edition (DSM-IV) criteria for alcohol addiction [2]. Alcohol addiction incidence varies from 1% to 5% according to WHO for developed and developing countries (including Greece) [3-5]. The financial cost of alcohol addiction at the level of health loss, social and financial burden is so big that alcohol addiction has become a public health priority [6]. Overall, in Europe alcohol abuse and addiction is held responsible for 1.8 million deaths (that is, 3.2% of the total causes of mortality) and 58.3 million (4%) of the total of life years in incapacity [7]. In Greece, epidemiology of alcohol use has shown changes from the traditional way of drinking alcohol and according to a 2004 study, the average consumption is 11.39 litres per capita; this gives Greece 10th position amongst the 26 European countries, with the index of harmful use being 2 [8]. There is also evidence showing that alcohol consumption is very common in Greece, since 1 in 4 adults drinks often (at least 10 times in the last month) and that excessive alcohol consumption (5 or more drinks at every session during the last month) happens 1 time in 10. This type of consumption (binge drinking) is very common in young adults (18 to 24 years old), with male predominance (1:5) [9].

Because of the importance of alcohol abuse/addiction in public health and its association with a wide spectrum of medical, social and psychological problems, early detection at the onset of abuse is very important. This requires specific tools to help diagnosis such as CAGE (named for an acronym of its four questions) and the Alcohol Use Disorders Identification Test (AUDIT) [10]. The aim of this study is to validate AUDIT in a Greek population. AUDIT detects alcohol abuse/addiction and is used in many studies as well as being a screening instrument for specific populations. AUDIT was created by a working group of the WHO by choosing questions that discriminate high risk drinkers in a six nations study [11].

## Materials and methods

AUDIT consists of 10 questions scored individually from 0 to 4. A total score of >8 is an indication of alcohol abuse, a score of >15 indicates serious abuse/addiction whilst a score between 8 and 10 is an indication of being at risk, according to the authors [10,11]. The questionnaire contains 10 questions; three questions on use, four on dependence and three questions about problems related to use.

AUDIT was translated in Greek from the English original by bilingual psychiatrists and back-translated from Greek to English by another bilingual psychiatrist.

A total of 218 subjects took part (128 males, 90 females). Of them, 109 subjects (75 males and 34 females) fulfilled DSM-IV criteria for alcohol addiction and were recruited from alcohol treatment units. The questionnaire was completed by care workers and psychiatrists with long experience in administering psychiatric rating scales. Average daily alcohol consumption of the alcohol-addicted subjects was 250 g during the last 6 months. All had a history of addiction of 5 years or more.

A further 109 individuals (53 males, 56 females) were used as healthy controls. These had no physical or psychiatric disorder and did not fulfil the DSM-IV criteria for alcohol addiction. Controls were recruited from hospital medical and nursing staff, teachers and manual workers. Data on sex, age and family status were collected.

The average age of the sample was 40.71 (± 11.34). In all, 39.1% were unmarried and 14.7% separated.

## Results

Reliability of internal consistency (Cronbach α index) was 0.7288 for the controls and 0.7989 for the patients (Table 1). Omitting the first question increased the Cronbach α index, but this increase was small and changes to the questionnaire were not required (Table 1). There was no statistically significant age difference between the alcoholic sample and the controls.

**Table 1:** Alcohol Use Disorders Identification Test (AUDIT) reliability analysis (Cronbach α)

AUDIT questions	Control group (no of cases = 109)		Alcoholics group (no of cases = 109)	
		
	Corrected item/total correlation	α if item deleted	Corrected item/total correlation	α if item deleted
How often do you have a drink containing alcohol?				
How many drinks containing alcohol do you have on a typical day when you are drinking?	0.4342	0.7664	0.5902	0.7698
How often do you have six or more drinks on one occasion?	0.5118	0.7562	0.5832	0.7705
How often during the last year have you found that you were not able to stop drinking once you had started?	0.5841	0.753	0.6739	0.7565
How often during the last year have you failed to do what was normally expected from you because of drinking?	0.4719	0.7657	0.5771	0.768
How often during the last year have you needed a first drink in the morning to get yourself going after a heavy drinking session?	0.5993	0.7605	0.4724	0.7835
How often during the last year have you had a feeling of guilt or remorse after drinking?	0.5536	0.7583	0.5076	0.7771
How often during the last year have you been unable to remember what happened the night before because you had been drinking?	0.7058	0.7509	0.5588	0.7709
Have you or someone else been injured as a result of your drinking?	0.3985	0.7739	0.2828	0.807
Has a relative or friend or a doctor or another health worker been concerned about your drinking or suggested you cut down?	0.5245	0.7543	0.3183	0.7974
Reliability coefficient (N = 10)	α = 0.7828		α = 0.7989	

The healthy controls had significantly lower average scores in the questionnaire 3.8 (± 3.61) (t test *P *< 0.001) when compared to the average scores of alcohol-dependent individuals (26.69 (± 8.39); Table 2). Controls scored lower average scores in all 10 questions of the AUDIT questionnaire (Table 3).

**Table 2:** Mean of age and Alcohol Use Disorders Identification Test (AUDIT)

		**Age**	**AUDIT**
Control group	Mean	39.66	3.79
	N	109	109
	SD	12.80	3.60
Alcoholic group	Mean	41.75	26.68
	N	109	109
	SD	9.61	8.39
Total	Mean	40.71	15.24
	N	218	218
	SD	11.34	13.15

SD, standard deviation.

**Table 3:** Means of Alcohol Use Disorders Identification Test (AUDIT) questions (Q) between alcoholic and control group

		**N**	**Mean**	**Standard deviation**
AUDIT Q1	Alcoholic group Control group	109 109	3.59 2.24	7.83 1.08
AUDIT Q2	Alcoholic group Control group	109 109	2.90 3.21	1.22 5.59
AUDIT Q3	Alcoholic group Control group	109 109	3.22 3.67	1.22 6.47
AUDIT Q4	Alcoholic group Control group	109 109	3.02 1101	1.44 4.78
AUDIT Q5	Alcoholic group Control group	109 109	2.19 7.339E-02	1.55 4.24
AUDIT Q6	Alcoholic group Control group	109 109	1.85 6.422E-02	1.75 3.40
AUDIT Q7	Alcoholic group Control group	109 109	2.94 1.28	1.48 4.3
AUDIT Q8	Alcoholic group Control group	109 109	2.27 8.257E-02	1.45 3.63
AUDIT Q9	Alcoholic group Control group	109 109	1.45 2.38	1.63 7.56
AUDIT Q10	Alcoholic group Control group	109 109	3.21 1.65	1.26 7.26

The questionnaire's sensitivity calculated for answers ≥ 8 was 0.98 (107/109) and its specificity 0.94 (101/109).

In the control sample, males had a higher average AUDIT score (t test *P *< 0.001) when compared to females (5.02 ± 4.10 vs 2.64 ± 2.62) (Table 4). However, in the sample of alcohol-dependent individuals there is no difference between male and female average AUDIT score (males 26.36 ± 8.57 vs 27.41 ± 8.06 females) (Table 5).

**Table 4:** Sex and Alcohol Use Disorders Identification Test (AUDIT) score in controls

	**Mean**	**N**	**Standard deviation**
Male	5.018*	53	4.10
Female	2.642*	56	2.61
Total	3.798	109	3.60

*Student t test *P *< 0.01.

**Table 5:** Sex and Alcohol Use Disorders Identification Test (AUDIT) score in alcoholic group

	**Mean**	**N**	**Standard deviation**
Male	26.36*	75	8.56
Female	27.41*	34	8.00
Total	26.68	109	8.39

*Student t test *P *> 0.05.

Age had a negative correlation with AUDIT score in the alcohol-dependent population (Pearson's *P *< 0.005, r = -221). Family status did not appear to influence the questionnaire in both alcoholic subjects and controls (ANOVA *P *> 0.005).

## Discussion

AUDIT has a high level of internal consistency and high reliability and validity in relation to clinical diagnosis. It detects 97% of patients and with a cut-off point set at 10 points has high sensitivity and specificity. There is no need to alter the cut-off point in relation to gender and the higher scores associated with male sex in the control population can be attributed to other parameters. Patients addicted to alcohol and other psychoactive substances have a wide range of needs that should be addressed if health services aim to provide the level of care needed [10]. The appropriate care should be based on prevention/education, recognition/detection, treatment [11,12] and follow-up.

Prevention of the physical and psychiatric complications of alcohol abuse/addiction is one of the main pillars on which care should be based, and this is closely associated with early detection/recognition of problem-drinkers. On the issue of early diagnosis, AUDIT can offer substantial help since it is quick and easy to use as well as reliable. AUDIT can be of help in screening populations at risk and patients with comorbid mental disorders [13]. This comorbidity can include schizophrenic disorders, mood disorders, personality disorders and other major psychiatric disorders [14,15].

AUDIT can also be used in Emergency Departments in order to aid differential diagnosis between psychotic symptoms or symptoms induced by alcohol abuse and addiction, since it is established that alcohol problems are underdiagnosed in psychiatric emergency assessments [13,16]. Using AUDIT could help in the screening of patients presenting to General Hospitals and prompt referral to psychiatric services and alcohol units [16]. This would lead to improved outcomes, since late detection of abuse/addiction is associated with poor therapeutic outcomes. This is the case not only regarding alcohol addiction but for concomitant physical illness as well [17]. Additionally, it is established that alcohol addiction is comorbid with psychosis, anxiety, emotional and personality disorders as well as attention deficit disorder and hyperactivity [18].

AUDIT is a reliable and sensitive instrument and is widely used in Europe and the rest of the world. It has been translated into many languages. It is used not only in primary care but in inpatient settings as well [19-22].

## Conclusion

Alcohol abuse/dependence, apart from being a major health issue, is also related to a wide spectrum of medical, psychiatric and social problems. Early detection and diagnosis is vital for prevention and treatment of these alcohol related problems. Early detection is not an easy task. Patients often have difficulties in admitting the level of their daily alcohol consumption. Using questionnaires that can detect covert forms of alcohol addiction is very important. AUDIT, having been validated in a Greek population, can now be used by teams and programs working in the field of alcohol addiction as it has been proven to be a useful and reliable instrument.

## Competing interests

The authors declare that they have no competing interests.

## Authors' contributions

GM and GD designed the study. GM and AD wrote the paper. EP, AT, DB, CC collected the data and statistically analysed them. LL had the overall supervision of the study.

## References

[B1] World Health Organization (2003). The World Health Report.

[B2] American Psychiatric Association (1994). Diagnostic and Statistical Manual of Mental Disorders.

[B3] Dawson DA (2000). Alcohol consumption, alcohol dependence, and all-cause mortality. Epidemiology and prevention. Alcohol Clin Exp Res.

[B4] Grant BF, Hanson DS, Stinson FS, Dawson DA, Chou SP, Ruan WJ, Pickering RP (2004). Prevalence, correlates and disability of personality disorder in the United States: results from the national epidemiologic survey on alcohol and related conditions. J Clin Psychiatry.

[B5] World Health Organization (2004). Global status report on alcohol 2004.

[B6] Dawson DA, Grand BF, Stinson FS, Chou PS (2006). Estimating the effect of help-seeking on achieving recovery from alcohol dependence. Addiction.

[B7] World Health Organization (2001). Alcohol in the European region – consumption, harm and policies.

[B8] Rehm J, Room R, Brink W Van den, Jacobi F (2005). Alcohol use disorders in EU countries and Norway: an overview of the epidemiology. European Neuropsychopharmacology.

[B9] Kitsos G, Kontogeorgiou K, Bafi I, Karahaliou K, Kokkevi A (2006). Alcoholic beverages: Use and addiction. Annual Report for Drugs and Alcohol 2005.

[B10] Saunders JB, Aasland OG, Babor TF, de la Fuente JR, Grand M (1993). Development of the Alcohol Use Disorders Identification Test (AUDIT): WHO collaborative project on early detection of persons with harmful alcohol consumption – II. Addiction.

[B11] Babor TF, Higgins-Biddle JC, Saunders JB, Monteiro M (2001). AUDIT – the Alcohol Use Disorder Identification Test: guidelines for use in primary health care.

[B12] Garnick DW, Horgan CM, Chalk M (2006). Performance measures for alcohol and other drug services. Alcohol Res Health.

[B13] Kessler RC, Crum RM, Warner LA, Nelson CB, Schulenberg J, Anthony JC (1997). Lifetime co-occurrence of DSM-III-R alcohol abuse and dependence with other psychiatric disorders in the National Comorbidity Survey. Arch Gen Psychiatry.

[B14] Dervaux A, Bayle FJ, Laqueille X, Bourdel M-C, Leborgne M, Olie J-P, Krebs M-O (2006). Validity of the CAGE questionnaire in schizophrenic patients with alcohol abuse and dependence. Schizophrenia Res.

[B15] Bowden-Jones O, Iqbal MZ, Tyrer P, Seivewrit N, Cooper S, Judd A, Weaver T (2004). Prevalence of personality disorder in alcohol and drug services and associated comorbidity. Addiction.

[B16] Foster J, Heather N (2005). Brief interventions for alcohol problems in hospital settings. Nurs Times.

[B17] Monras M, Mondon S, Ortega L, Gual A (2005). Alcoholism in the general hospital: 4 years mortality and hospitalization. Med Clin (Barc).

[B18] Brady KT, Verduin ML, Tolliver BK (2007). Treatment of patients comorbid for addiction and other psychiatrc disorders. Curr Psychiatry Rep.

[B19] Gache P, Michaud P, Laundry U, Accieto C, Arfaoui S, Wenger O, Daeppen JB (2005). The Alcohol Use Identification Test (AUDIT) as a screening tool for excessive drinking in primary care: reliability and validity of a french version. Alcohol Clin Exp Res.

[B20] Gomez Arnaiz A, Conde Martela A, Alberto Aquiar Bautista J, Manuel Santana Montesdeoca J, Jorrin Moreno A, Betancor Leon P (2001). Diagnostic usefulness of Alcohol Use Disorders Identification Test (AUDIT) for detecting hazardous alcohol consumption in primary care settings. Med Clin (Barc).

[B21] Conde Martel A, Gomez Arnaiz A, Aquiar Bautista JA, Santana Montesdeoca JM, Jorrin Moreno A, Suarez Ortega S (2000). Diagnostic usefulness of the questionnaire "Alcohol Use Disorders Identification Test" (AUDIT) to detect conditions associated with alcohol in hospitalized patients. An Med Interna.

[B22] Conigrave KM, Saunders JB, Reznik RB (1995). Predictive capacity of the AUDIT questionnaire for alcohol-related harm. Addiction.

